# Active self-motion control and the role of agency under ambiguity

**DOI:** 10.3389/fpsyg.2023.1148793

**Published:** 2023-04-20

**Authors:** Anne-Laure Rineau, Bruno Berberian, Jean-Christophe Sarrazin, Lionel Bringoux

**Affiliations:** ^1^ONERA, Information Processing and Systems Department (DTIS), Salon-de-Provence, France; ^2^CNRS, ISM, Aix Marseille Univ, Marseille, France

**Keywords:** self-motion perception, multisensory integration, agency, discrimination task, predictive coding

## Abstract

**Purpose:**

Self-motion perception is a key factor in daily behaviours such as driving a car or piloting an aircraft. It is mainly based on visuo-vestibular integration, whose weighting mechanisms are modulated by the reliability properties of sensory inputs. Recently, it has been shown that the internal state of the operator can also modulate multisensory integration and may sharpen the representation of relevant inputs. In line with the concept of *agency*, it thus appears relevant to evaluate the impact of being in control of our own action on self-motion perception.

**Methodology:**

Here, we tested two conditions of motion control (active/manual trigger versus passive/ observer condition), asking participants to discriminate between two consecutive longitudinal movements by identifying the larger displacement (displacement of higher intensity). We also tested motion discrimination under two levels of ambiguity by applying acceleration ratios that differed from our two “standard” displacements (i.e., 3 s; 0.012 m.s^−2^ and 0.030 m.s^−2^).

**Results:**

We found an effect of control condition, but not of the level of ambiguity on the way participants perceived the standard displacement, i.e., perceptual bias (Point of Subjective Equality; PSE). Also, we found a significant effect of interaction between the active condition and the level of ambiguity on the ability to discriminate between displacements, i.e., sensitivity (Just Noticeable Difference; JND).

**Originality:**

Being in control of our own motion through a manual intentional trigger of self-displacement maintains overall motion sensitivity when ambiguity increases.

## 1. Introduction

Whether for simple or more complex tasks, accurate perception of one’s own motion is crucial. It is now widely accepted that accurate perception of self-motion requires integrating a variety of information provided by both environment-centred (e.g., optic flow) and body-centred cues (e.g., vestibular, proprioceptive inputs and motor output; Cheng and Gu, 2018; Cullen, 2019). During the last decades, the human brain has been credited with developing adaptive mechanisms that contribute to an optimal integration of multisensory cues by combining redundant and complementary inputs accounting for stimulus characteristics (Ernst and Banks, 2002; Alais and Burr, 2004; Ernst and Bülthoff, 2004; Fetsch et al., 2013). Seminal works stressed the importance of sensory reliability of inputs in multisensory integration for self-motion perception (Gu et al., 2008; Morgan et al., 2008; ter Horst et al., 2015).

As promoted by the concept of *Active sensing* (Kveraga et al., 2007; Schroeder et al., 2010; van Atteveldt et al., 2014), it has been recently suggested that multisensory processing does not depend solely on the nature of sensory inputs, but also on the motor and attentional contexts of an action (Donohue et al., 2015). A growing body of studies recently investigated perceptual responses in situations where an external event (stimulus) is the result of an intentional action (van Kemenade et al., 2016; Arikan et al., 2017; Straube et al., 2017). Sensory integration during self-generated actions has been found to be modulated at both physiological (Hughes et al., 2013) and behavioural (Bays et al., 2006) level compared to the processing of the same sensory inputs generated by an external system. Yet most studies exploring self-motion perception are limited to “passive” stimulations, i.e., where motion is not self-generated (Cheng and Gu, 2018). So far, visuo-vestibular integration for motion perception has not been investigated through the prism of intentional action.

However, the link between intention and perception has been widely studied within the theoretical framework of agency. Indeed, the sense of agency describes the subjective feeling associated with controlling one’s own actions and, through these actions, events in the outside world (Haggard and Tsakiris, 2009; Chambon and Haggard, 2012). Agency is largely explained through a comparator model (CM) that describes internal computational predictive mechanisms of human action control (Frith et al., 2000; Blakemore et al., 2002). Interestingly, previous work has highlighted the dependence of the vestibular system on this model. Indeed, a decrease in response of the VO (*vestibular only*) neurons was specifically observed when the efference copy due to active motion was in agreement with current sensory inputs, both for rotation (Roy and Cullen, 2004) and translation (Carriot et al., 2013). Specifically, it has recently been reported that the active vs. passive internal state distinction at an early stage of integration may be a source of modulation in the computation of motion (Gu, 2018; Brooks and Cullen, 2019; Cullen and Wang, 2020; Cullen and Zobeiri, 2021). There is, however, strikingly little information on the consequences of active versus passive states on self-motion perception. In this context, our study explored the impact of the intentional nature of an action triggering a visuo-vestibular stimulation on self-motion perception, as seen through the prism of agency.

Yon and Frith (2021) recently argued that intentional action comes with prior knowledge (e.g., prediction) that could be used to optimise perception in an uncertain world Yon and Frith (2021). In addition, recent work demonstrates that being active (in terms of motor control of the action) potentiates the integration of relevant cues at the audio-visual level (van Kemenade et al., 2016; Arikan et al., 2017). It can therefore reasonably be hypothesised that being in control of an action may optimise the integration of the different sensory inputs relevant to the task at hand. Here, we speculate that the agentive context of an action may help reduce uncertainty by promoting multisensory integration of relevant inputs. Our hypothesis is consistent with the fact that action can be considered as a powerful way to reduce uncertainty, since it allows better prediction of outcomes (Yon et al., 2018). We therefore speculate that this reduction of uncertainty during sensory integration would help refine the distinction of motions whose characteristics would slightly vary. Thus, being intentionally active during a perceptual task may be particularly valuable in situations with a high level of sensory ambiguity.

The present study sought to explore how being active might impact the perception of one’s own motion at different levels of sensory ambiguity. Specifically, we aimed at investigating to what extent having control over one’s own motion help distinguish it from an externally generated motion under uncertainty. To that purpose, we used a two-alternative forced-choice (2AFC) discrimination task adapted from previous studies on audition (Reznik et al., 2015; Paraskevoudi and SanMiguel, 2021). In this task, the participants had to compare two displacements and identify which was larger. To compare active versus passive motion, two different conditions were presented. In the active condition, the first displacement was intentionally triggered by the participant whilst the second was externally generated (that is, without any participant-intentional action). In the passive condition, both displacements were externally generated. The first displacement had a fixed acceleration value, and the second displacement varied around this value. Two levels of ambiguity were introduced into the discrimination task by manipulating acceleration ratios between the two movements. We expected better perceptual discrimination between movements when participants intentionally triggered the first displacement than when both displacements were externally generated, particularly under a high level of ambiguity between movements.

## 2. Materials and methods

### 2.1. Participants

Twenty participants (12 men, 8 women, M_age_ = 27, SD = 5, age range: 20–32 years) took part in the experiment. This sample size was defined, using a power analysis based on a comparable study based on sound discrimination (Paraskevoudi and SanMiguel, 2021). Participants were recruited in the population of students and engineers of the ONERA center of Salon-de-Provence. They were all naïve to the purpose and hypotheses of the study. None of them reported vestibular or other sensory issues (all had corrected-to-normal vision), nor any history of motion or cybersickness. The French CERSTAPS ethics committee approved the experiment (IRB00012476-2021-23-06-119), and participants gave their informed consent prior to the experiment, in accordance with the 1964 Declaration of Helsinki.

### 2.2. Apparatus

**Figure 1:**
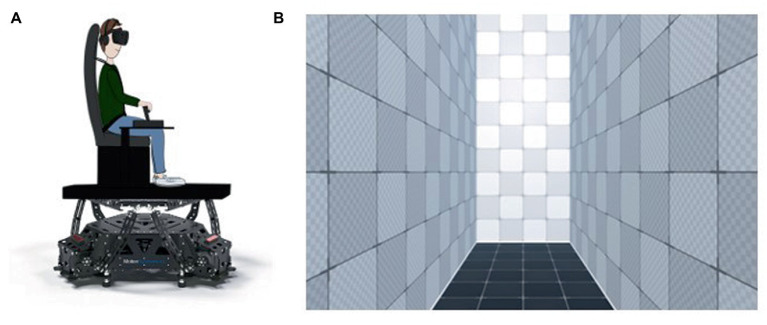
**(A)** Schematic representation of the setup, **(B)** Virtual environment.

The physical motion was generated via a mobile platform (Motion Systems PS-6TM-550_©_; Figure 1A). The visual dynamic environment was simulated using a virtual reality headset (Varjo VR-3 Pro_©_). It consisted in a virtual textured corridor (provided by the Unity3D game engine) 2 m wide x 5 m high x 6.25 m deep (Figure 1B). All visual events allowing the participant to situate himself/herself in the trials (trial start signal, choice gauge, movement announcements, responses) occurred at a helmet distance of 3 m in the virtual corridor. Participants’ position in the virtual environment was individually calibrated via a pair of cameras (SteamVR Lighthouses 2.0_©_) installed at the top two corners of the wall facing the platform. The image centre was adjusted to each participant’s eye level. All actions, choices and responses of participants were generated by a dual throttle controller (Thrustmaster HOTAS Warthog TM) and a button placed at the handle extremity. To mask any possible sound information from the platform, earphones (Turtle Beach Stealth 350VR headset) were used to produce constant white noise throughout trials.

### 2.3. Experimental task and stimuli

Participants were subjected to forward translations of a fixed duration of 3 s and with a triangular acceleration profile (1.5 s of acceleration and 1.5 s of deceleration). This same profile was applied at the visual level so that participants advanced through the virtual scene by scrolling the textured corridor congruently and synchronously to the platform. Based on this, they had to perform a two-alternative forced-choice (2AFC) discrimination task, identifying which displacement, the first (standard) or second (comparison), was larger.

Participants performed the comparison task at two levels of intensity, i.e., standard displacements. The first was the minimum stimulation under which the platform could generate all the comparison pairs, i.e., 0.012 m.s^−2^, considered here as the low level of intensity. The second was set at 0.030 m.s^−2^, close to the maximum limit (distance to be covered) of the system, considered here as the high level of intensity. In both cases, we made sure that the standard stimuli were perceptible by the participants, i.e., above threshold (determined via a detection task performed the day before). From these standard values, we increased or decreased the acceleration rate of the second comparison displacement. Thus, the comparison displacement was of variable value, being more or less large, stronger or weaker than the standard displacement, depending on the differences applied. These differences were of 0, ±0.002, ±0.004, ±0.006, ±0.008, ±0.010 m.s^−2^ and + 0.012 m.s^−2^. Thus, whatever the level of intensity, the differences in acceleration rate between the first standard (fixed) displacement and the comparison (variable) displacement were identical. However, for a given difference, the ratio to the standard reference differed depending on the current intensity level. Indeed, by keeping the same comparison values between the two intensity levels, we can claim to have generated two conditions in which the differences were more or less marked from a relative point of view (i.e., a difference of 0.002 m.s^−2^ is relatively more marked for a 0.012 m.s^−2^ standard stimulation than for a 0.030 m.s^−2^ standard stimulation). This configuration thus yielded two conditions differing in terms of ambiguity (difficulty), providing a high level of ambiguity for the high level of intensity, and a low level of ambiguity for the low level of intensity.

In addition, the participants performed the task under two conditions of motion control, i.e., passive (observer) versus active (manual trigger). In the active condition, participants manually chose the intensity level for the next trial. By confirming his/her choice via a button press, he/she had control over the first displacement, whereas the second displacement was generated automatically. In passive trials, both displacements were automatically generated. Thus, 4 types of trials were considered: Active-High (AH), Active-Low (AL), Passive-High (PH) and Passive-Low (PL). Each block of 48 trials allowed all test types (AH, AL, PH, PL) and comparison values to be presented once in a randomised fashion. Each participant performed 6 blocks, thus representing 288 pseudo-randomized trials, for a total duration of approximately 2h10min. A mandatory break was scheduled in the middle of the session, but the participant was free to take a break at the end of each block.

### 2.4. Procedure

Participants were first provided with the experimental objectives and instructions and signed the consent form. Then, they were strapped into seats on the mobile platform. The two-alternative forced-choice (2AFC) discrimination task started after a calibration process and the completion of a training block of about 15 trials.

**Figure 2:**
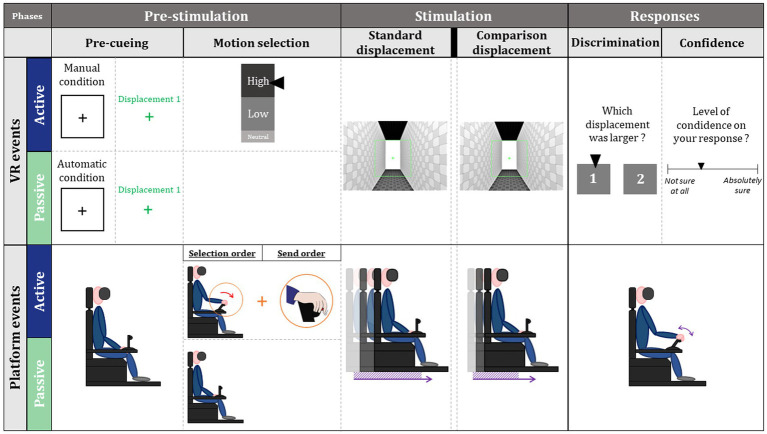
Schematic representation of the different phases of a trial.

During the 2AFC discrimination, each trial had three main phases: a pre-stimulation phase, a stimulation phase, and a response phase (Figure 2).

The pre-stimulation phase began with a one-second signal onset (black frame and central cross) that indicated whether the trial would be externally generated (passive) or intentionally generated (active). In active condition, the participant was also kept informed of the number of trials of each type (low or high) remaining to be generated by him or her. Then, the first displacement was announced during 1 s. In active condition, participants were then asked to choose a level of intensity for the next trial through a cursor to be positioned on a visual gauge, using a throttle (Figure 2: *motion selection*). Then, they press a button on the throttle to generate the first displacement which started without latency after the disappearance of the gauge. In the passive condition, as previously designed in a similar task, the interval between visual cue and the standard displacement onset was randomly selected from the participants’ distribution of press times (Paraskevoudi and SanMiguel, 2021). In addition, participants were not informed of the type of intensity selected in this passive condition.

Then, they were entering the stimulation phase during which the two displacements (i.e., standard and comparison displacements, lasting 3 s each) were successively produced (Figure 2: *standard and comparison displacements*). Importantly here, the comparison displacement was announced and always externally generated after visual pre-cueing. The time interval between pre-cueing and the start of the comparison displacement was randomly distributed from each participant’s distribution of press times. The stimulation phase ended with a red ending signal (END) presented for 1 s.

Then, participants entered a response phase during which they first identified and then confirmed with the throttle one of the two displacements as larger (displacement of higher intensity; Figure 2: *discrimination*). Next, they had to respond using a continuous scale (analogical from 1 to 5) from *“I am not sure at all”* to *“I am absolutely sure,”* to assess their confidence on motion discrimination (Figure 2: *confidence*).

The trial ended and the platform was moved back from the final to the initial position following a fixed time smooth animation trajectory during 3 s for the participants to be ready for the next trial. Videos of the events that take place during the trials, from the point of view of the virtual environment, are available in supplemented data.

### 2.5. Data analysis

The proportion of responses perceiving the second displacement as larger was calculated for each condition, according to the different comparison values. This was used to fit psychometric curves for each participant and condition with a normal cumulative function via the *quickpsy* package of R (version 4.0.0). We used this package since it has been specifically developed for this type of analysis (Linares and López-Moliner, 2016) and recently used in a similar task (Paraskevoudi and SanMiguel, 2021). The lower asymptote of the psychometric function that corresponds to the gamma parameter of the fitting model was set to 0. The upper asymptote (i.e., lambda) which corresponds to the lapse rate was set to 0.001. These fitting parameters have previously been used in other 2-AFC discrimination tasks (Paraskevoudi and SanMiguel, 2021) and enabled us to generate fitting models with the most satisfactory Akaike information criterion for our data. The Akaike Information Criterion (AIC) is a mathematical method for evaluating how well a model fits the data from which it was generated. An AIC score is assigned based on the relative amount of information lost by a given model. Thus, the less information a model loses, the lower the score, and the higher the quality of the model.

Two variables were extracted from the psychometric curves for each participant in each condition. First, the Point of Subjective Equality (PSE) corresponding to the value at which the comparison displacement is judged statistically equal to the standard displacement, which is used to express a potential perceptual bias across conditions. Indeed, a shift in PSE relative to the Point of Objective Equality (i.e., the point of physical equality between the two displacements, here 0 m.s^−2^) reflects a biassed estimate of perceived motion intensity. A higher PSE indicates that the standard displacement is perceived as larger. Indeed, if the PSE is positive, the participant judged the two displacements as identical when the comparison displacement had a slightly higher acceleration rate than the standard displacement (by the value of the PSE). The standard displacement is then perceived as a displacement that has a higher acceleration rate than its actual acceleration rate. The PSE corresponds to the alpha value of the model. Second, the Just Noticeable Difference (JND) was extracted to establish the discrimination sensitivity between the two displacements. This corresponds to the minimum gap between stimuli for perceiving a difference between displacements. Thus, the lower it is, the better the performance. The higher it is, the more difficult for the participant to discriminate differences between motions. Therefore, and in accordance with Weber’s law, a greater JND is expected as the intensity increases. The JND corresponds to the beta value of the model.

In addition, the confidence level each participant reported for their responses was recorded for all conditions. Higher confidence indicates less uncertainty in the participant’s judgement on the current task. Additional metacognition analyses were conducted based on these confidence levels, enabling the *M-ratio* and *meta-d*’ variables to be explored. These analyses assessed the participants’ ability to translate their performance back into their confidence levels. Indeed, the *meta-d*’ reveals a degree of cognitive sensitivity. It is the ability of the participant to adapt his/her confidence to his/her performance (i.e., to give high confidence scores when he is right and to give low confidence scores when he is wrong). The higher it is, the better the cognitive sensitivity of the participant. The *M-ratio* is a ratio of the *meta-d’* to the *d’*. *d’* represents the performance on the task and is not transcribed here since the JND is our reference of discrimination performance. Besides, the analyses showed that the results behave in the same way for these two parameters. Metacognitive efficiency was computed for each participant based on confidence scores, in each condition separately, using the *metaSDT* package (Craddock, 2018) in the R environment.

All these variables (i.e., PSE, JND, levels of confidence, *M-ratio* and *meta-d*’) were analysed using a repeated measures ANOVA combining two factors: Agency condition (Active versus Passive) and level of ambiguity (Low versus High). Effect sizes were estimated using partial eta-squared (η^2^_p_). All statistical analyses were performed using R (version 4.0.0). Analysis codes are available by the authors without under request and without undue reservation.

## 3. Results

### 3.1. Psychometric curves

**Figure 3:**
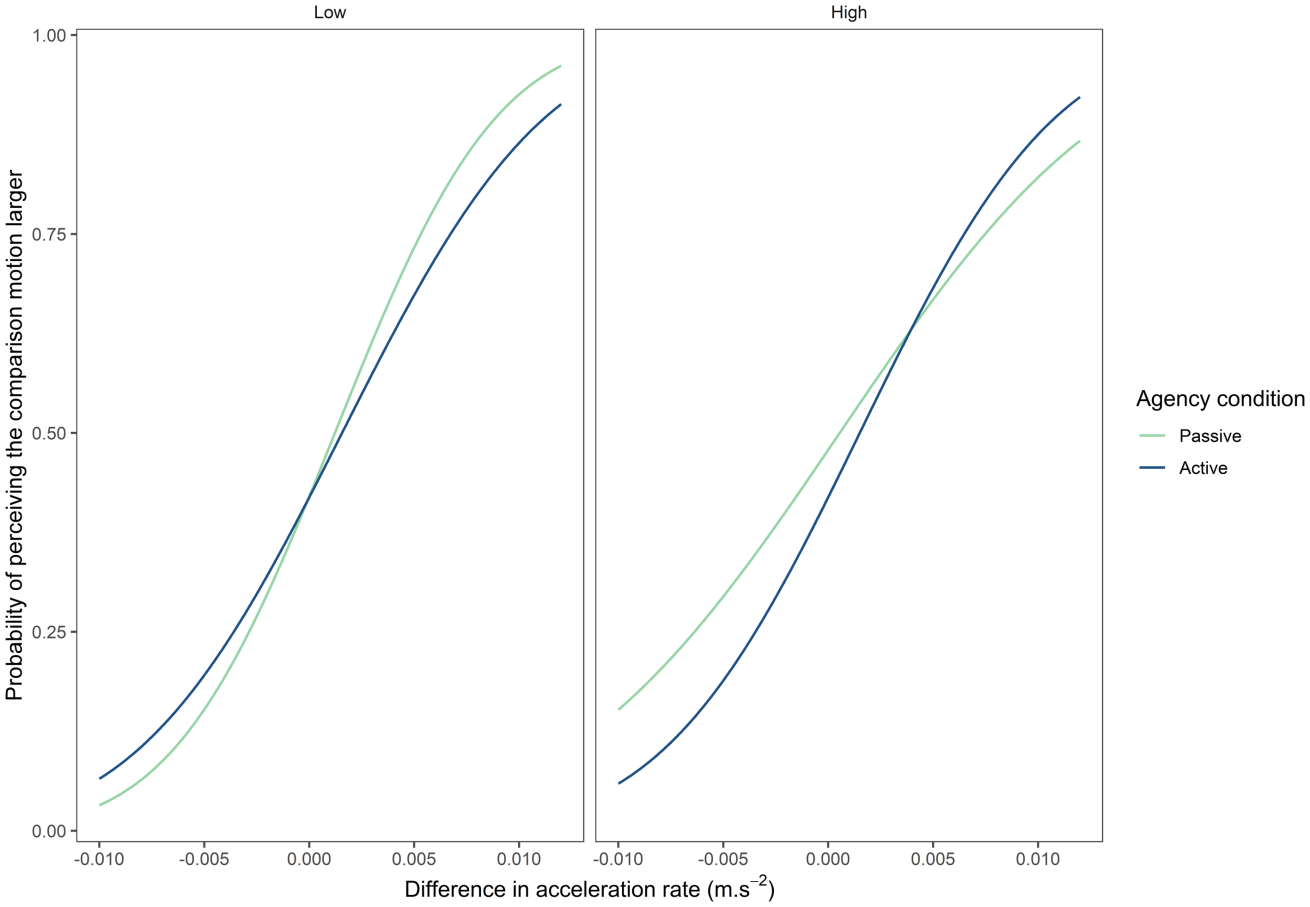
Psychometric curve depending on level of motion ambiguity and agency condition.

Visual inspection of the psychometric curves from the discrimination task (Figure 3) reveals a comparable slope for both levels of ambiguity in the self-generated motion condition (active). However, the slope appears to become stiffer under high ambiguity in the externally generated motion condition (passive). More strikingly, there appears at first glance to be a difference between the active and the passive condition under both levels of motion ambiguity (low *vs.* high). To corroborate these observations, statistical analyses of the PSE and JND extracted from each individual curve were subsequently performed. Indeed, to assess whether theses parameters differed across agentive conditions and levels of ambiguity, we performed a repeated-measures ANOVA evaluating the influence of the agentive condition (automatic *vs.* manual) and the level of ambiguity (high *vs.* low). We applied the correction of Bonferroni for post-hoc comparisons.

**Figure 4:**
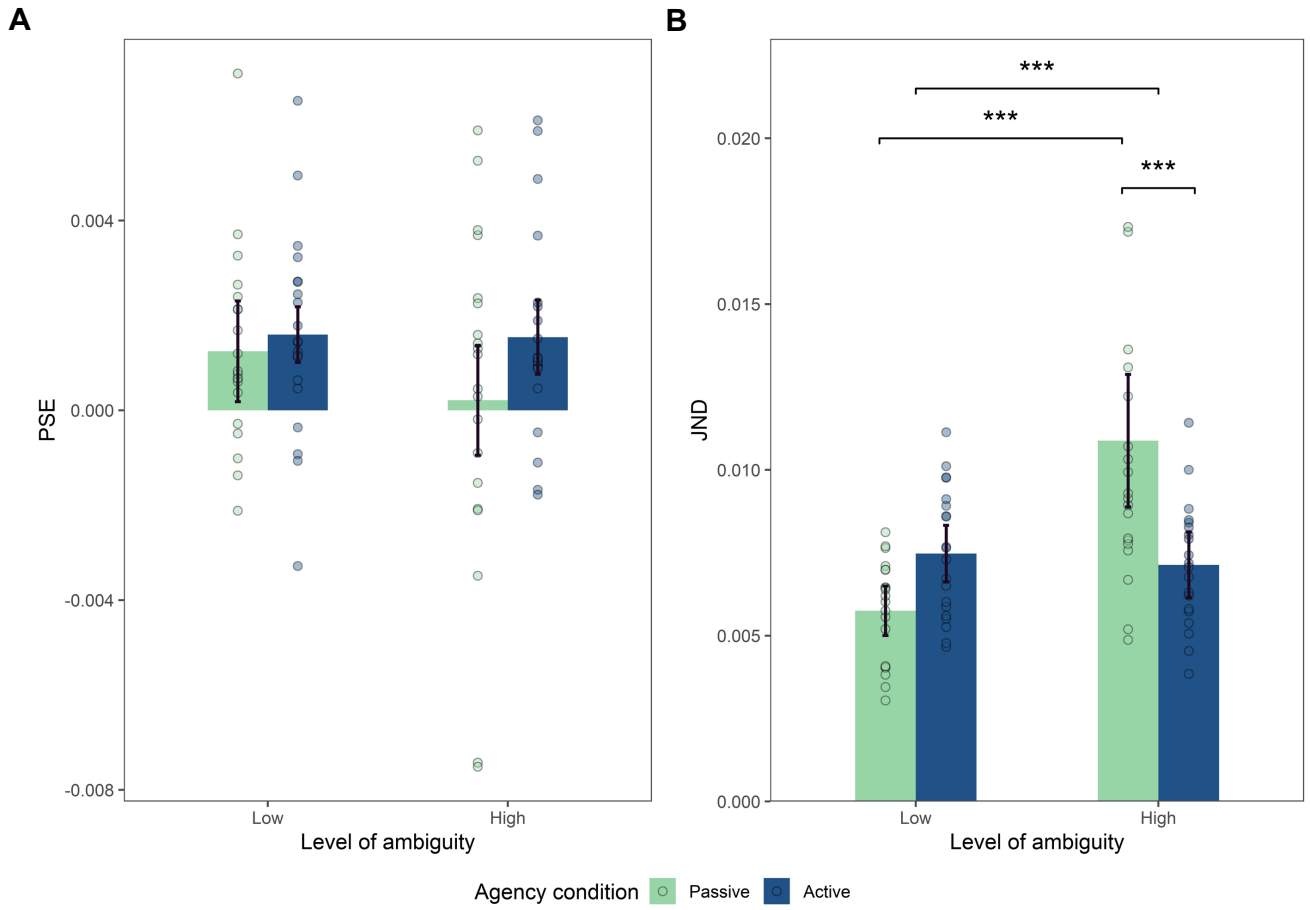
**(A)** Mean of PSE, **(B)** Mean of JND. Significant effect of interaction between motion ambiguity and intensity on JND (*p*<0.001), with post-hoc comparison showing lower JND for the active than for passive high ambiguity condition (one-tailed paired samples post-hoc *t*-test; *p*<0.001) and significantly higher JND for the passive high ambiguity than for the passive low ambiguity condition (one-tailed paired samples *post hoc t*-test; *p*<0.001). Error bars in Figures represent the within-subject confidence intervals, calculated using the *summarySEwithin* function in *R*.

The analysis conducted on PSE did reveal a significant main effect of the agentive condition (*F*_(1,19)_ = 6.90, *p* = 0.017, η^2^*_p_* = 0.27) with a higher PSE in the agentive condition (M_A_ = 1.7.10^−3^, M_P_ = 0.9.10^−3^, SD_A_ = 0.002, SD_P_ = 0.003). However, no effect of the level of motion ambiguity was revealed (F_(1,19)_ = 1.76, *p* = 0.2; Figure 4A) nor were any interaction effects (*F*_(1,19)_ = 0.76, *p* = 0.395). In addition, we analysed whether any of the PSEs differed significantly from zero. T.tests revealed that PSEs differed from zero for AL (*t* (19) = 3.30, *p* = 0.004), PL (*t* (19) = 2.71, *p* = 0.014), and AH (*t* (19) = 3.12, *p* = 0.006) conditions, whilst it was non-significant for the PH condition (*t* (19) = 0.27, *p* = 0.79).

In contrast, the analysis conducted on JND did not reveal a main effect of the agentive condition (*F*_(1,19)_ = 2.71, *p* = 0.12; Figure 4B). Moreover, it revealed a main effect of the level of ambiguity (*F*_(1,19)_ = 19.06, *p* < 0.001, η^2^_p_ = 0.50) with a higher JND for the high level of ambiguity (M_L_ = 0.007, M_H_ = 0.009, SD_L_ = 0.002, SD_H_ = 0.004). Importantly, a significant interaction between the two factors (*F*_(1,19)_ = 19.07, p < 0.001, η^2^_p_ = 0.50). The Bonferroni corrected post-hoc analysis revealed that JND was lower for intentionally generated motions (active) than for externally generated motions (passive) under high ambiguity (M_AH_ = 0.007, M_PH_ = 0.011, SD_AH_ = 0.002, SD_PH_ = 0.005, *t* (19) = −4.27, *p* < 0.001, *d* = 0.98). In addition, JND was significantly higher for high *vs*. low motion ambiguity in the passive condition (M_PL_ = 0.006, M_PH_ = 0.011, SD_PL_ = 0.002, SD_PH_ = 0.005, *t* (19) = 6.16, *p* < 0.001, d = 1.22).

### 3.2. Confidence and metacognition

The analyses on level of confidence values revealed a significant effect of agentive condition (*F*_(1,19)_ = 5.77, *p* = 0.027, η^2^_p_ = 0.23). Participants were more confident when they intentionally generated the motions (M_A_ = 3.63, M_P_ = 3.56, SD_A_ = 1.14, SD_P_ = 1.14). However, no effect of level of ambiguity was found (*F*_(1,19)_ = 1.47, *p* = 0.24) nor were any interaction effects with agentive condition (*F*_(1,19)_ = 2.16; *p* = 0.16).

**Figure 5:**
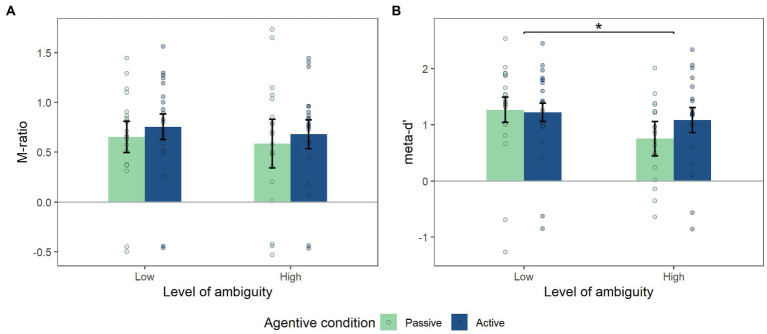
**(A)** Mean of M-ration, **(B)** Mean of meta-d’. significant effect of level of ambiguity on the meta-d’ variable (*p*<0.05). But no effect of agentive condition. Error bars in Figures represent the within-subjects confidence intervals, calculated using the *summarySEwithin* function in *R*.

Complementary metacognition analyses were conducted on these levels of confidence to assess the extent to which the participant’s reported level of confidence is correlated with his/her performance. The *M-ratio* analysis did not reveal any effect of level of ambiguity (*F*_(1,19)_ = 0.65, *p* = 0.43) or of agentive condition (*F*_(1,19)_ = 1.24, *p* = 0.28), and nor were any interaction effects on this variable (*F*_(1,19)_ = 0.002, *p* = 0.92; Figure 5A). In contrast, analysis did reveal an effect of level of ambiguity on the *meta-d’* variable with a lower *meta-d’* in the high level of ambiguity (M_L_ = 1.24, M_H_ = 0.92, SD_L_ = 0.85, SD_H_ = 0.76, *F*_(1,19)_ = 7.87, *p* = 0.011, η^2^_p_ = 0.29). However, no effect of agentive condition was revealed (*F*_(1,19)_ = 1.56, *p* = 0.22; Figure 5B). Even though we did not find any interaction effect between the two factors (ambiguity, agentive condition), a trend was noted (*F*_(1,19)_ = 3.13, *p* = 0.09).

## 4. Discussion

According to Bergson’s thinking (1896), voluntary action is linked to perception. Thus, understanding what governs being in control could help to elucidate human perception. Exploring the intentional nature of action may shed light on the mechanisms underlying perception. Recently, it has been proposed that action control could be a way to sharpen representation of expected outcomes (Yon et al., 2018; Jagini, 2021). Based on these premises, the present study explored the role of action control in the perception of one’s own motion at different levels of sensory ambiguity. Indeed, we designed a discrimination task enabling us to manipulate both the ambiguity of pairs of displacements and the voluntary nature of the action. Performance on the discrimination task as well as participants’ reported confidence in their perception of the displacements were considered for each trial. We expected better perceptual discrimination between displacements when participants intentionally triggered the first movement, especially under a high level of motion ambiguity.

Analysis of the psychometric curves and of confidence levels highlighted three main results. First, we found better sensitivity (lower JND) in the active condition than in the passive condition, specific to a high level of ambiguity. Second, we observed an effect of agentive condition (higher PSE in the manual condition) but not of the level of ambiguity on PSE (perceptual bias). Third, we observed a general effect of agentive condition, with a higher level of confidence under the agentive condition but no effect of interaction with level of ambiguity.

The two main parameters (PSE and JND) obtained from participants’ psychometric curves indicate the way they performed the task. The PSE represents the value to be added to the standard displacement for the comparison displacement to be perceived as equal. It indicates how that standard displacement is perceived since a difference in perceptual bias (PSE) between conditions represents either a perceptual attenuation or an enhancement of the first displacement relative to its physical reality. We did not observe any effect of level of ambiguity. In other words, the first displacement was perceived identically under both levels of ambiguity. Also, we did not find any interaction effect between ambiguity and agentive conditions. In contrast, we did find a general effect of the agentive condition with higher PSE in manual condition. Overall, participants tended to enhance the first displacement in the agentive condition. In recent years, the control of action has been associated with both phenomena of attenuation and enhancement of the related sensory processing. It seems here that the context of action and its sensory consequences themselves play indeed a role in the underlying mechanisms. For example, previous studies suggest that this may depend on the intensity of the sensory consequences (Reznik et al., 2015; Paraskevoudi and SanMiguel, 2021). In our study, two levels of intensity have been manipulated (*“High”* and *“Low”*). However, they can be both considered as rather low displacement intensities (as reported by the participants). We can assume here that having control over the first displacement increases in both cases its perception to better perceive it and compare it to the second one. The second parameter (JND) is particularly informative on sensitivity of discrimination between displacements, representing the value at which a difference between the two displacements is perceived: the lower the JND, the better the discrimination performance. Better sensitivity (lower JND) was observed in the active condition with a high level of ambiguity than in the passive condition. Taken together, these results suggest that having control over the first displacement lead to higher stability of mechanisms enabling its discrimination from the second when ambiguity vary. Therefore, being active leads to better discrimination of controlled information (first displacement) from uncontrolled information (second displacement) when ambiguity increases.

It should be noted that previous studies using the same type of discrimination task observed different effects of the active condition on these two parameters (Reznik et al., 2015; Paraskevoudi and SanMiguel, 2021). For example, Paraskevoudi and SanMiguel (2021) found reduced perceived intensity (i.e., perceptual bias) for self-generated sounds when presented at supra-threshold intensities (high level), but which increased when presented at near-threshold intensities (low level). Also, the authors found no difference in discrimination sensitivity (i.e., JND). The fact that we extended the aforementioned paradigm to cover several new and important aspects could explain such differences.

First, in our study, participants had control over both the level of intensity they wanted to work at and the timing of the first displacement (the pair of displacements starting as soon as they confirmed their choice). Therefore, the agentive condition strongly mobilises the participant’s intention in the forthcoming comparison pair, rather than in continuous control of the displacements. We decided to add this intentional selection because the choice between different possible actions strongly influences the experience of control and agency (Barlas and Obhi, 2013; Barlas et al., 2017; Barlas and Kopp, 2018). One could hypothesise here that it comes from a higher predictability of event following the choice (i.e., regarding both timing and intensity of the first displacement). In addition, we speculate that the predictive mechanisms involved in this agentive situation lie more in the predictability and attentional commitment related to the participant’s intention. Indeed, it is now established that agency is associated with a better commitment to the task (Caspar et al., 2016), with the mobilisation of attentional mechanisms (Wen and Haggard, 2018). Since the participant’s intention was always respected, we speculate that being in intentional control of the first displacement allowed for a better integration of the related information. More precisely, according to the comparator model, the agentive situation refines the comparative action-consequence mechanisms linking action to its consequences. We can speculate that this serves a better integration of sensory inputs related to the action. Besides, it has recently been suggested that action may help sharpen sensory integration of relevant sensory outcomes (Schroeder et al., 2010; Yon et al., 2018). In addition, we did observe a general stability of both parameters (i.e., perceptual bias and sensitivity) when ambiguity vary. Indeed, we note that sensitivity of discrimination between the two levels of ambiguity remained constant in the active condition whereas it decreased in the passive condition (higher JND) when ambiguity increased. Thus, we speculate that intentional control led to smaller prediction error on this first displacement, allowing better detection of the prediction error gap on a second uncontrolled displacement, even when ambiguity increased. In our high ambiguity condition, the differences in the second displacement were more difficult to perceive (in terms of ratio from the standard displacement). Rather than demonstrating better discrimination in ambiguous situations under the agentive condition, our results tend to show a reduction in performance under the passive condition when the level of ambiguity increases. Therefore, a lower error signal in the agentive condition appears to promote stability and consistency in integrative performance when ambiguity increases. In this case, the prediction error difference between the first controlled displacement and the second uncontrolled displacement remains sufficient to maintain discrimination. In contrast, a fully passive situation generates higher prediction error, preventing the detection of smaller deviations from the first displacement during the second. Thus, our results highlight the impact of the decrease in prediction error in an agentive situation when there is a match between intentional control and the consequences. The fact that the difference from the passive situation is observed under more ambiguous conditions strengthens this hypothesis.

Second, we considered a far more complex multisensory stimulation than in previous studies, which mainly considered unimodal input (mainly bip sound). Recent studies suggest that predictive mechanisms engaged during an action promote the binding of sensory inputs relevant to the task at hand (van Kemenade et al., 2016; Arikan et al., 2017; Straube et al., 2017). Since our results differ from those of previous studies conducted under a specific single sensory stimulation, we also speculate that action may shape sensory consequences differently depending on the amount of task-relevant information available. Also, it is undeniable that future studies would clarify the part of each of underlying mechanisms in such agentive condition (i.e., attention, prediction, predictability, choice).

In addition, we performed an analysis of the confidence levels and of the metacognition of the participants. Having observed a general effect of being in control on confidence levels, but no interaction effect with the level of ambiguity, we decided to complement our analysis by considering metacognition variables. The *meta-d*’ variable was used to evaluate the correlation between the participant’s confidence in his/her performance and the reality of the performance itself (Maniscalco and Lau, 2012; Fleming and Lau, 2014). Therefore, when this score decreases, the participant’s metacognitive performance decreases. An effect of the level of ambiguity on metacognitive performance is observed: as the level of ambiguity increases, metacognitive performance decreases. Interestingly, we observe a trend towards interaction effects with the agentive condition. Associated with the graphical representation of these results (Figure 5B), the metacognitive performance can be seen to strongly decrease in the passive condition, compared to the active condition.

One limitation here is the number of trials compared to other studies using much shorter stimulations (increasing the number of trials using our multisensory stimulations would have made the study too long and onerous for the participants). A greater number of trials might highlight this trend towards maintaining metacognitive performance in the agentive condition. However, it is interesting to note that the agentive nature of the stimulation could promote not only the mechanisms of multisensory integration but also the mechanisms underlying metacognitive performance.

## 5. Conclusion

To our knowledge, our study is the first to extend the notions of both agency and ambiguity management to human motion perception. Its main contribution lies in showing that being in control of one’s motion is beneficial when faced with ambiguous situations. Our conclusions are strengthened by the fact that participants did not perceive themselves as performing better in the high-ambiguity active condition. Such a difference in the management of ambiguous situations should be further explored to better understand the perception of an observer versus an operator, particularly for critical situations. This would provide answers in key areas such as aeronautics and the automotive industry. Thus, there is clearly a need to better understand the underlying integrative mechanisms involved according to the operator’s level of control.

## Data availability statement

The raw data supporting the conclusions of this article will be made available by the authors under request and without undue reservation.

## Ethics statement

The studies involving human participants were reviewed and approved by The French CERSTAPS ethics committee (IRB00012476-2021-23-06-119). The patients/participants provided their written informed consent to participate in this study.

## Author contributions

A-LR, BB, J-CS, and LB: study conception and design, interpretation of results, and draft manuscript. A-LR: data collection and analysis preparation. All authors contributed to the article and approved the submitted version.

## Funding

This work was supported by APR Grants (DAR 1107) from the French National Space Research Centre (CNES).

## Conflict of interest

The authors declare that the research was conducted in the absence of any commercial or financial relationships that could be construed as a potential conflict of interest.

## Publisher’s note

All claims expressed in this article are solely those of the authors and do not necessarily represent those of their affiliated organizations, or those of the publisher, the editors and the reviewers. Any product that may be evaluated in this article, or claim that may be made by its manufacturer, is not guaranteed or endorsed by the publisher.
